# Sub‐Terahertz Memristor Switches Using MoS_2_ by Liquid–Liquid Interface Assembly

**DOI:** 10.1002/advs.75909

**Published:** 2026-06-29

**Authors:** Tomás Mingates, Mohamed E. Ghatas, Jonas Deuermeier, Adam G. Kelly, Joseph Neilson, Jonathan N. Coleman, Luís Mendes, João C. Vaz, Sérgio Matos, Luca Lucci, Antonio Clemente, Zdeněk Sofer, Luís M. Pessoa, Elvira Fortunato, Rodrigo Martins, Asal Kiazadeh

**Affiliations:** ^1^ CENIMAT|i3N Department of Materials Science School of Science and Technology NOVA University Lisbon and CEMOP/UNINOVA Caparica Portugal; ^2^ INESC TEC – Institute for Systems and Computer Engineering Technology and Science and FEUP – Faculdade de Engenharia da Universidade do Porto Porto Portugal; ^3^ School of Physics CRANN & AMBER Research Centers Trinity College Dublin Dublin Ireland; ^4^ Instituto de Telecomunicações Lisbon and Instituto Politécnico De Leiria Leiria Portugal; ^5^ Instituto de Telecomunicações Lisbon and Instituto Superior Técnico Universidade De Lisboa Lisbon Portugal; ^6^ Instituto de Telecomunicações University Institute of Lisbon (ISCTE‐IUL) Lisbon Portugal; ^7^ Université Grenoble Alpes CEA‐LETI Grenoble France; ^8^ Department of Inorganic Chemistry University of Chemistry and Technology Prague Prague Czech Republic

**Keywords:** 2D materials, 6G, memristor, MoS2, programmable metasurfaces, RF switches, terahertz communications

## Abstract

This study demonstrates the first application‐ready radio‐frequency (RF) switches based on memristors fabricated via electrochemical exfoliation and liquid–liquid interfacial assembly. This 2D layer deposition method produces uniform, low‐defect bilayer molybdenum disulfide (MoS_2_) nanosheet networks without the high temperatures or hazardous gases typical of chemical vapor deposition, providing a low‐cost, environmentally friendly route toward CMOS‐compatible integration. The resulting devices exhibit robust unipolar resistive switching, simplifying biasing and lowering power consumption compared to bipolar solutions. Devices show reproducibility with 10^4^ s retention and 100‐cycle endurance. RF characterization confirms reliable operation across 10–110 GHz with low insertion loss (0.42–0.9 dB), isolation above 18 dB, and an intrinsic cut‐off frequency of ∼5.4 THz. The viability for integration into energy‐efficient wireless communication platforms is assessed by simulation of a 24 × 24 elements reconfigurable intelligent surface. High gain (>21.6 dBi) and efficient beam steering (−60°–60°) across the 26.8–29.1 GHz band demonstrate the utility of these novel non‐volatile RF switches in next‐generation mmWave communication, including 5G/6G and satellite systems.

## Graphical abstract

This work introduces application‐ready sub‐terahertz memristor switches fabricated from electrochemically exfoliated MoS_2_ nanosheets assembled at a liquid–liquid interface. The devices exhibit robust unipolar resistive switching, low insertion loss, and high isolation across 10–110 GHz. Integration into reconfigurable intelligent surfaces demonstrates efficient beam steering, positioning these scalable and CMOS‐compatible switches as a promising route toward 6G wireless platforms.

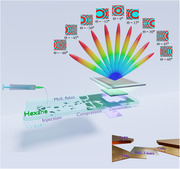

## 1 Introduction

Reconfigurable intelligent surfaces (RIS) have been considered as a pivotal enabling technology, with the potential to enhance communication network quality and energy efficiency within forthcoming generations of wireless communication. This technology consists of an extensive array of unit cells with programmable elements that locally control its electromagnetic properties, enabling precise tailoring of the metasurface's overall scattering response. The integration of RIS into an infrastructure facilitates directional control of wireless signals, which can be advantageous to various applications, including but not limited to autonomous vehicles, industrial automation, and Internet of Things (IoT) networks in smart cities [1, 2]. In several cases, the interaction of RIS technology with communication systems has been demonstrated to achieve the desired objectives. These objectives include overcoming undesired obstacles, the enhancement of the coverage area by providing line‐of‐sight paths, and improving signal strength. RIS technology enables advanced and emerging features, such as user‐specific beamforming and enhanced network security by avoiding suspicious directions. In addition, RIS can be deployed at the cell edges to reduce inter‐cell interference, improve network performance, and increase both spectral efficiency and data rate transmission.

An RF switch is a device that manages radio frequency signals, enabling the selection between multiple signals or the connection and disconnection of signal paths within a circuit. These switches play a vital role in directing signals among various components, such as amplifiers, mixers, and filters, allowing the use of multiple frequency bands as needed in mobile communications, reconfigurable radio systems, IoT applications, and phased‐array networks and will play a key role in future communication technologies [3, 4, 5, 6, 7]. In particular, they are crucial in active RIS technology.

The predominant challenge with all existing technologies is the power consumption required to maintain and/or change the RF switch state [5, 8]. Non‐volatile RF resistive switches (nv‐RS) offer distinct advantages by retaining their resistance state even when the power supply is removed, distinguishing them from traditional RF switches [9, 10, 11, 12]. In IoT systems, where devices often operate on limited power resources, nv‐RS contribute significantly to energy efficiency by eliminating the need for continuous power to maintain their state. Furthermore, nv‐RS enhances the reliability of critical applications, ensuring that configurations remain intact during power disruptions. Recent advancements have shown that memristor‐based RF switches as nv‐RS can effectively manage high‐frequency signals with minimal insertion loss and excellent isolation (see Table S1) [7, 13, 14, 15, 16, 17, 18, 19, 20, 21, 22, 23]. A comprehensive comparison with alternative RF switching technologies is presented in Table S2 [24, 25, 26, 27, 28, 29]. The secret to these excellent figures of merit lies within the filamentary resistive switching mechanism: Shrinking the device area for minimal off‐capacitance does not compromise the on‐resistance, because the latter is primarily determined by the filament diameter.

Besides the power consumption issue, additional hardware impairments of various RF switch technologies specific to RIS applications exist and have recently been reviewed [30]. At high frequencies, such as sub‐THz bands, miniaturization becomes critical for programmable metasurfaces in general and RIS technology in particular, as unit cells shrink to a microscale size (typically *λ*/2) and space constraints become more severe. Discrete electronic devices such as varactors, RF MEMS switches, and PIN diodes are typically flip‐chipped onto radiating surfaces fabricated using printed circuit board technologies. However, at terahertz frequencies, heterogeneous integration of packaged semiconductors becomes highly challenging due to the sub‐wavelength dimensions of the radiating elements and the stringent requirement for high efficiency (i.e., minimal insertion and integration losses). Memristors have a typical footprint of only a few micrometers or below. Low‐temperature thin‐film processing (not requiring a specific substrate material) allows fabrication directly on the RIS substrate, eliminating the need for soldering and traditional component packaging. Some of the most promising memristor‐based RF switches have been fabricated with an active layer based on 2D materials (see Table S1). However, typically chemical vapor deposition (CVD) is used to deposit the 2D layers which involves the use of hazardous gases, which is a cost‐driving factor due to the gas handling and not environmentally friendly [31, 32, 33].

In this article, we present the solid‐state molybdenum disulfide (MoS_2_) memristors by liquid–liquid (LL) assembly of electrochemically (EC) exfoliated nanosheets. In the following, these devices will be referred to as electrochemical exfoliation and liquid–liquid interfacial assembly (EC‐LL) MoS_2_ memristors. A previous study has demonstrated the fabrication of memristors using a liquid–liquid interface technique, where a monolayer of MoS_2_ was deposited onto an indium tin oxide (ITO) bottom electrode, with eutectic gallium–indium (EGaIn) gel serving as the top electrode [34]. Notably, this device was not patterned, and the bottom contact was common across the device area. It is expected that once patterning is introduced, the resistive switching characteristics will change significantly. Moreover, these memristors utilized liquid‐phase exfoliation for nanosheet formation, which typically results in nanosheets with a broad thickness distribution. In contrast, the electrochemical exfoliation method employed in our study produces ultrathin MoS_2_ nanosheets, leading to higher‐quality films with greater uniformity [35]. Electrochemical exfoliation also eliminates the need for toxic organic solvents and can be efficiently and sustainably scaled for large‐area applications, unlike the commonly used CVD [31, 32, 33]. Additionally, our memristors incorporate application‐friendly electrodes (electron beam evaporation of metals and pattering by photolithography), further enhancing their practicality. The memristors operate via a unipolar switching scheme. One of the primary benefits of unipolar switches is their simplicity in operation; they require only a single polarity of voltage to toggle between states, which reduces the complexity of circuit design. Our devices show performances comparable to state‐of‐the‐art RF switches, exhibiting isolation levels greater than 18 dB, which, while lower than those reported for hexagonal boron nitride (hBN)‐based devices (∼35 dB) and MoS_2_‐based switches (∼25 dB), fall within acceptable limits for practical mmWave applications. Additionally, the insertion loss ranges between 0.42 and 0.9 dB, aligning closely with several reported values in the literature (0.4 to 1 dB) [7, 17, 18]. From the equivalent circuit model, we extract a cut‐off frequency of approximately 5.4 THz. This value falls within the reported range for similar 2D‐material‐based RF switches (ranging from 4.7 to 129 THz) [7, 17], further validating the suitability of our devices for high‐frequency applications. Previous studies primarily focus on switch performance, often neglecting the impact of cycle‐to‐cycle (C2C) and device‐to‐device (D2D) variations, and many investigations remain at the proof‐of‐concept level. In this study, we investigate these parameters thoroughly, establishing a more robust understanding of resistive switching technology in RF applications. Additionally, full‐wave simulations demonstrate that these high‐performance miniaturized RF switches facilitate the design of a new class of energy‐efficient reconfigurable intelligent surfaces for 6G applications.

## 2 Results and Discussion

### 2.1 The EC‐LL MoS_2_ Memristor Device

**FIGURE 1:**
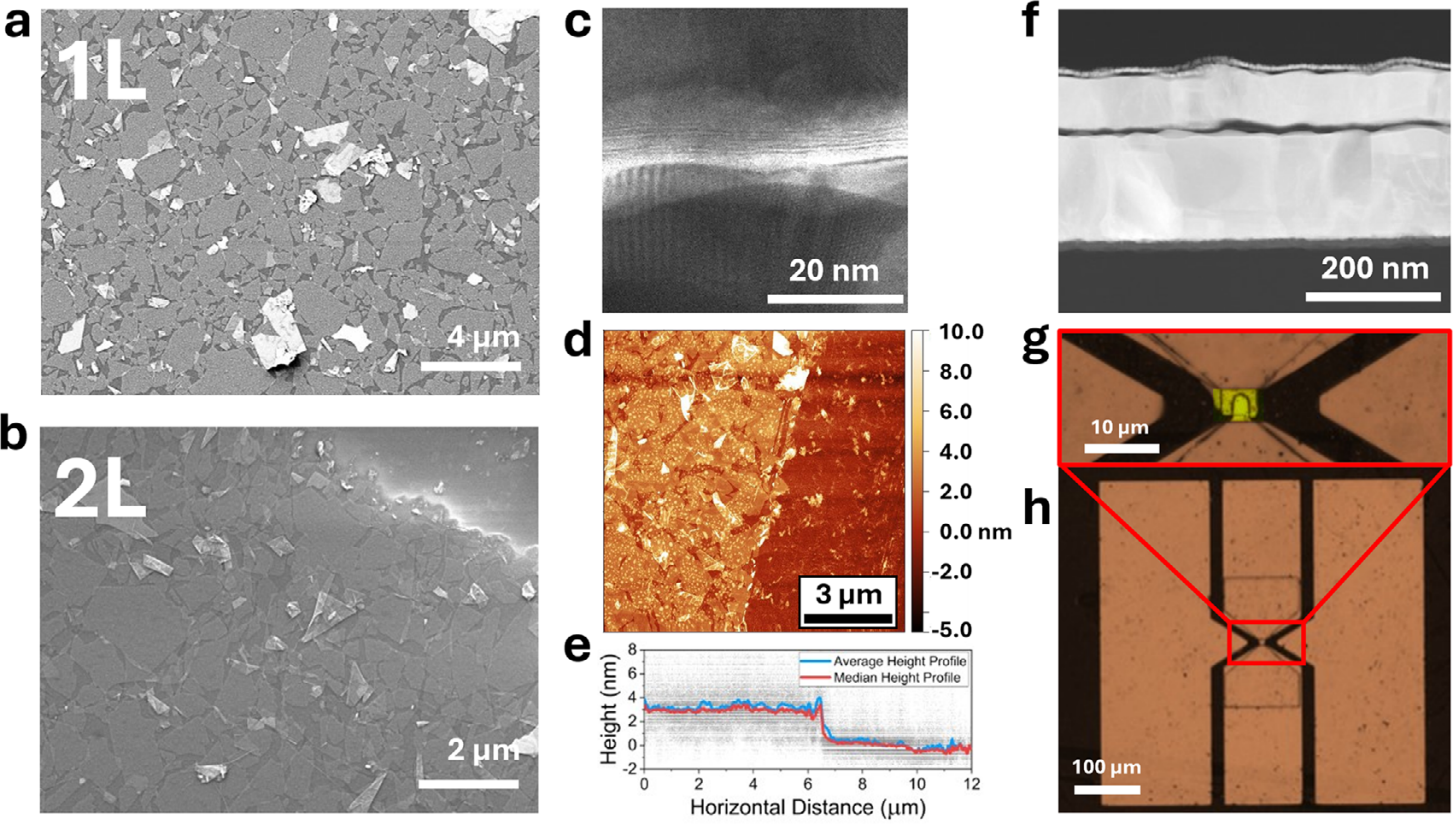
Morphological characteristics of the device. (a) SEM of 1L MoS_2_ film (1 deposition step). (b) SEM of 2L MoS_2_ film (2 deposition steps). (c) STEM of Au/MoS_2_(2L)/Cr/Au device cross‐section. (d) AFM of MoS_2_ showing 2L deposition with a step from the substrate. (e) Average and median of all horizontal profile scan lines, aligned by the step. (f) STEM of Au/MoS_2_(2L)/Cr/Au device cross‐section at a lower magnification. (g) Close‐up micrograph of the Au/MoS_2_(2L)/Cr/Au overlap device (color adjusted for enhanced visibility). (h) Micrograph of Au/MoS_2_(2L)/Cr/Au overlap device integrated with a Cu GSG CPW test fixture.

Networks of the electrochemically exfoliated molybdenum disulfide (MoS_2_) are fabricated using a liquid–liquid interfacial solution‐processing method (see Fabrication section). This technique uses the interface between the immiscible liquids water and hexane to assemble nanosheets into monolayer networks. When a 2D material ink is injected at this interface, nanosheets are trapped due to surface tension forces. A gradient in the water–hexane interfacial tension, induced by the isopropanol (IPA) solvent, drives Marangoni convection and compresses the nanosheets into a dense monolayer with predominantly edge‐to‐edge contacts. Stabilized by capillary forces, the assembled monolayer is then transferred to a substrate via vertical lifting. A layer‐by‐layer assembly approach was used to produce aligned and uniform multilayer nanosheet networks with low porosity, as shown in SEM images in Figure 1a,b of MoS_2_ one (1L) and two (2L) consecutive liquid–liquid interface assembly (LLIA) deposition steps, respectively, STEM image in Figure 1c and AFM images in Figure 1d, accompanied by a corresponding AFM line‐scan profile shown in Figure 1e. It is important to note that “2L” refers to two consecutive deposition steps rather than a strict bilayer structure. Due to the random placement of the MoS_2_ sheets during assembly, it is possible to obtain local single‐sheet coverage as well as additional stacking at the corners of overlapping sheets, which can lead to regions with three or more layers. Furthermore, because TEM captures a finite volume in depth, non‐flat and non‐uniform layers can create additional visual overlaps when viewed from the side, as observed in the STEM cross‐section in Figure 1c. The line‐scan profile indicates a 2L thickness of approximately 3–4 nm. Since a monolayer of MoS_2_ by CVD has a thickness of 0.7 nm [36], the additional material in our case derives from the polyvinylpyrrolidone (PVP) adsorbed at the surface. Compared to conventional liquid‐based deposition methods such as inkjet printing, spin‐coating, and spray‐coating, in which nanosheets fall randomly onto a substrate, the LLIA process offers significant improvements to nanosheet network morphology and electrical performance by enabling the formation of uniform, densely packed layers with less lateral space between the tiles. This feature enhances switching properties and decreases variability, due to fewer pinholes, making it ideal for next‐generation solution‐based and printed electronics, because the typically thick and often rough surfaces of printed layers can be conformally covered with nanosheets [37, 38, 39]. This is a key requirement for low device‐to‐device variability. However, the STEM image at lower magnification, Figure 1f shows varying electrode spacing and reveals areas of additional interfacial gaps. Rather than perfectly conforming to the underlying nanoscale topography, the continuous 2D MoS_2_ sheets bridge across local features. The EC‐LL MoS_2_ sheets apparently can bridge some of the valleys of the bottom electrode, creating additional vertical space between the metal electrodes. The impact of this feature on device performance is discussed further below.

For EC‐LL MoS_2_ device fabrication, photolithographic steps were employed to pattern the electrodes Figure S1. Two distinct designs were employed: a crosspoint configuration and an overlapped configuration, the latter illustrated in Figure 1g,h. A Ti (10 nm)/Au (60 nm) bottom electrode is coated with MoS_2_ via two consecutive LLIA depositions, followed by a Cr (2 nm)/Au (70 nm) top electrode. Ti and Cr were both used as adhesion promoters [15]. The devices were patterned with overlap areas of 4 and 25 µm^2^.

Devices embedded in ground‐signal‐ground (GSG) coplanar waveguide (CPW) test fixtures were implemented to enable RF characterization. Figure 1g,h shows optical microscope images of the EC‐LL MoS_2_ memristors integrated with 700 nm thick Cu waveguides on a fused silica substrate. To accommodate these fixtures, the memristor design was modified from the initial crosspoint structure to overlap with in‐line electrode pads. The waveguides provide a stable, controlled transmission path for RF testing, crucial for obtaining precise and reproducible measurements in high‐frequency electronic devices.

### 2.2 Switching Operation Scheme

**FIGURE 2:**
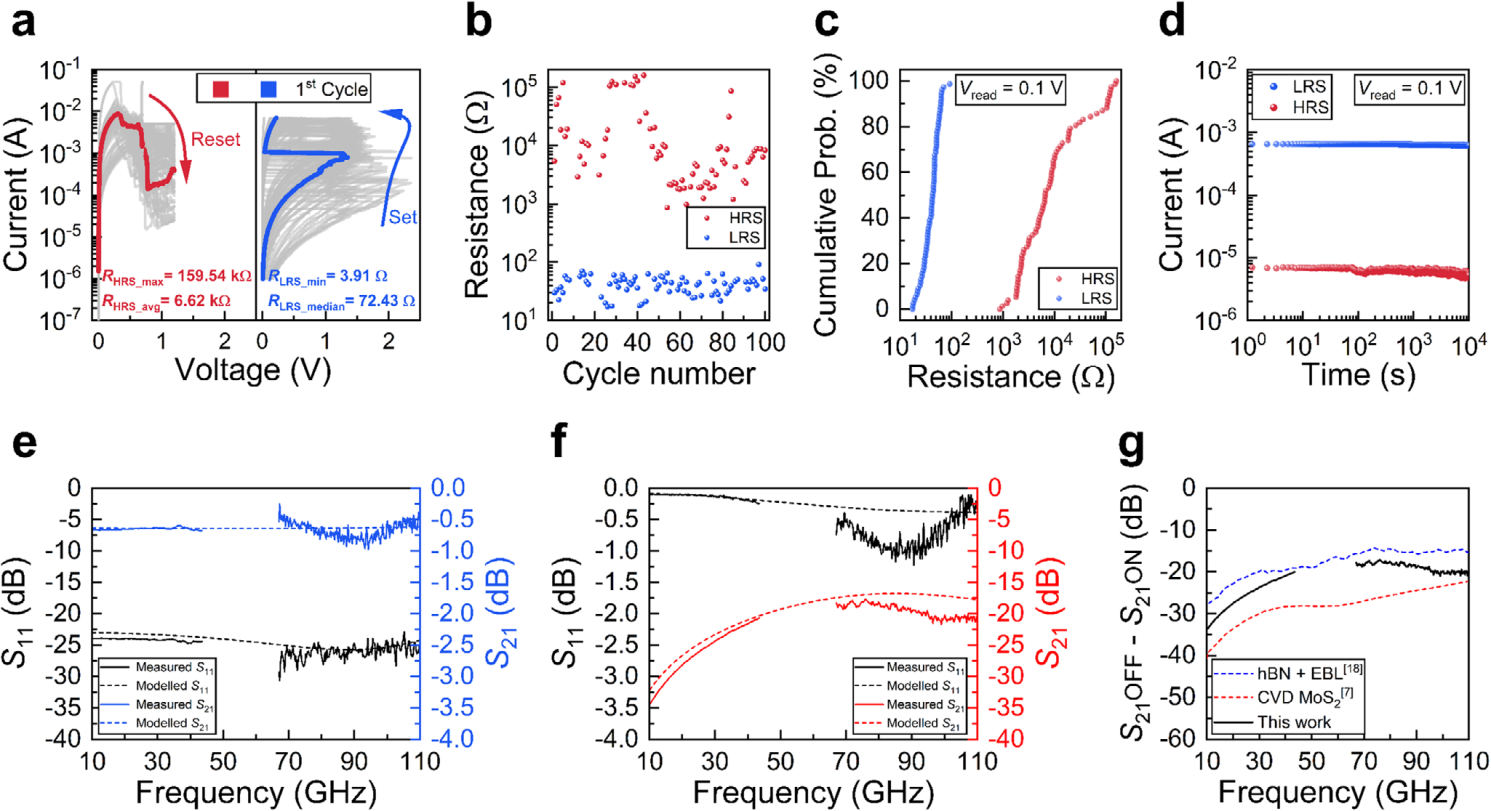
The *I*–*V* characteristics and RF performance measurements of the EC‐LL MoS_2_ memristor devices. (a) DC sweep cycle of endurance of overlapped devices: unipolar with current‐controlled set and voltage‐controlled reset (device area ≈ 4 µm^2^; 100 cycles). (b) Resistance values of HRS and LRS through the 100 consecutive cycles. (c) Cumulative probability distribution of the observed LRS and HRS throughout the 100 cycles. (d) Retention measurement of LRS and HRS states. (e,f) The de‐embedded measurement S‐parameters of the memristor in the LRS and HRS, respectively. The measurements are compared to the obtained simulation results of the equivalent circuit model by Keysight ADS 2020 software (g). The switching ratio of the EC‐LL MoS_2_ memristor compared with hBN employing EBL and CVD MoS_2_‐based memristors presented by Pazos et al. 2024 [18] and Kim et al. 2022 [7], respectively.

The devices show unipolar resistive switching behavior presented in Figure 2a, where the transition between high resistance state (HRS) and low resistance state (LRS) occurs under the forcing of a current. The set process in which the device changes from the HRS to the LRS is often driven by the migration of metal cations or other mobile species within the material, leading to the formation of a conductive filament, which is referred to as the electrochemical mechanism (ECM). In the present case, however, such long‐range ECM originating from the electrodes can be excluded. Although a localized Cr adhesion layer is present at one interface, the observed switching polarities are fully symmetric under bias reversal, which is inconsistent with an electrode‐asymmetric metallization process. This indicates that the resistive switching is more likely governed by an intrinsic defect‐mediated process within the active material or by a symmetrically activated interfacial/bulk mechanism associated with the Au contacts. A similar behavior of bipolar and unipolar coexistence was previously reported and attributed to thermally activated phenomena [36]. It is characteristic for thermally activated switching to operate symmetrically, which may also be called non‐polar, meaning that both the set and the reset will happen very similarly regardless of sweep polarity. This property is displayed in Figure S2, making them very versatile in terms of operation [18]. Furthermore, while PVP is an insulating polymer known to absorb moisture, our experimental data indicate that its residual presence does not fundamentally alter the device's operational mechanics or longevity. Specifically, our measured switching characteristics, retention, and endurance do not deviate meaningfully from standard devices fabricated with pure, CVD‐grown MoS_2_ as demonstrated in established works [7, 15, 36]. Because we observe no endurance degradation typically associated with moisture absorption, we conclude that hygroscopic effects are negligible in our specific architecture. Furthermore, the most established conduction mechanism for these devices—though still a subject of ongoing debate in the field—is the reversible inclusion of gold atoms into sulfur vacancies to form conductive filaments [40]. Because our devices achieve identically low ON‐state resistances compared to pristine MoS_2_ devices, it is highly probable filament formation process is very similar despite the PVP presence.

When the set process is executed by imposing a voltage, a power spike is induced that is limited only by the applied current compliance. However, due to the nanosecond discharge of statically stored charge from the setup's parasitic capacitance during filament formation, a standard compliance current often fails to arrest this transient fast enough. This abrupt power increase can unnecessarily stress the device and diminish the endurance [41]. To mitigate this, a current‐controlled sweep from 1 µA to 5 mA was implemented for the set operation as illustrated in Figure 2a. By sourcing a precise current, the driving voltage naturally collapses upon filament formation (snapback). While the stochastic nature of the microscopic filament formation still yields some natural variation in the final LRS, this self‐limiting method safely restricts the transient power dump and consistently achieves a low LRS. Notably, the resistance values extracted from devices with active areas ranging from approximately 1 to 100 µm^2^ exhibit no visible dependence of the LRS on the nominal device area, whereas the HRS remains substantially more dispersed (Figure S3). Such area‐insensitive LRS is inconsistent with homogeneous bulk conduction across the entire overlap region and instead indicates that the current is confined to highly localized conductive pathways. This observation provides further support for a filamentary or conductive‐point switching process, in which only a nanoscale fraction of the nominal junction area actively participates in the low‐resistance transport. The consistent resistive switching characteristics across the measured devices highlight the robustness of the switching mechanism (see Figure S4). As presented in Figure 2b,c, while the device exhibits good uniformity in the LRS, the HRS displays a noticeably broader variance. This difference is primarily driven by the inherent stochastic nature of the switching mechanism, specifically the unpredictable formation and partial rupture of one or multiple conductive filaments during the reset process. This specific statistical behavior, including the broader cycle‐to‐cycle variance in the HRS compared to the LRS, is a known characteristic of stochastic switching and is highly consistent with trends reported in similar nanoscale memristive systems by Pazos et al. 2024 [18]. However, from a practical RF operation standpoint, this DC variance in the HRS is largely negligible. Once the HRS resistance exceeds approximately 1 kΩ, the imaginary component, *C*_OFF_, dominates and ultimately dictates the device's overall impedance at high frequencies. Consequently, the variations within the LRS present a much more substantial factor for the RF circuit design than the broader DC fluctuations observed in the HRS. Regarding their retention time, non‐volatility was confirmed up to at least 104 s, depicted in Figure 2d.

The set operation is self‐limited by using current sweeps, but there is a risk of damaging the device by a premature set during the voltage‐controlled reset operation. For this reason, the reset procedure was divided into two steps. Each step used progressively lower compliance, with the first stage applying a voltage sweep from 0 to 0.7 V with a compliance of 50–80 mA, and the second stage applying a sweep from 0.7 to 1.2 V with a compliance of 1–5 mA, with adjustments made for device‐to‐device variations. In instances where a set event occurred during the second stage, the resulting LRS was not much distant from the standard level, hence creating no permanent damage to the device. Notably, two set events were observed during the first reset stage; however, these did not lead to catastrophic failure (more details in Note S1 and Figure S5). To explicitly handle this unipolar (non‐polar) switching challenge and ensure reliable device operation, we propose to utilize a “state check and repeat sweep” methodology in future system integration. Following a RESET sweep, the system applies a low‐voltage, non‐destructive read pulse to verify the device's resistance state. If the measured resistance falls below the target HRS threshold (indicating a premature SET event occurred), the system automatically triggers an additional RESET sweep. This closed‐loop verification process effectively mitigates the overlap issue, ensuring the device successfully reaches the intended state and driving the practical operational failure rate down to negligible levels.

While the demonstrated DC endurance of 100 cycles establishes the baseline reliability of the resistive switching mechanism, practical deployment of RF switches in commercial 6G RIS strictly necessitates significantly longer operational endurance. Currently, the cycle life of 2D‐material‐based memristors remains a broader challenge within the field. The primary limitation stems from the difficulty of repeatedly triggering and reverting the soft breakdown of the dielectric matrix without inducing permanent localized damage. In this study, a straightforward DC voltage sweep protocol utilizing an adaptive compliance current was employed to demonstrate facile integration with low‐complexity control electronics. However, to unlock the extended endurance required for 6G applications, transitioning from continuous DC sweeps to short‐duration pulsed voltage sequences is essential. Fast voltage pulsing allows for highly precise control over the delivered power, minimizing excessive Joule heating and power overshoots during the filament formation and dissolution processes. Recent literature demonstrates that implementing optimized pulsed voltage protocols can extend the endurance of comparable 2D memristive devices by orders of magnitude—from roughly a hundred cycles under standard DC sweeps to upward of 10 000 cycles [42]. Consequently, the 100 endurance cycles under DC shown in our work are not indicative for a generally limited endurance, but only a manifestation of the employed switching protocol, which was deliberately kept simple for facile integration with electronics of low complexity. Transitioning to high‐speed pulsed switching architectures represents the primary trajectory for the future development of these devices toward long‐term RIS deployment.

### 2.3 RF Characterization

RF measurements were conducted at various institutions, IT, Lisbon, and INESC‐TEC, Porto. Prior to these measurements, the batch of devices was programmed into LRS and HRS states. Four days and fourteen days after initial programming, the devices were re‐evaluated to verify state retention. The LRS remained unchanged, while the HRS stabilized to an even higher resistance, see Figure S6. The environmental stability of the MoS_2_ was also assessed. Some devices were fabricated by depositing the top electrode after a 5‐month storage in ambient conditions, on top of the previously deposited bottom electrode and MoS_2_ layer. The results demonstrate that the 2D layer maintains highly stable performance despite the aged MoS_2_ layer, see Figure S7.

The efficiency of the memristors was measured in terms of the scattering parameters (S‐parameters) for both LRS and HRS utilizing the vector network analyzer (VNA) and probe station, see Figure S8. RF measurements were performed combining short‐open‐load‐thru (SOLT) calibration with a second‐tier thru‐reflect‐line (TRL) step to de‐embed test fixture effects, see Note S2 and Figure S9 [43, 44, 45]. To illustrate the parasitic contributions of the substrate versus the intrinsic device performance, the raw S‐parameter data prior to de‐embedding for a representative device in the low‐frequency band (10–43.5 GHz) is compared with their de‐embedded results in Figure S10. Hence, the intrinsic characteristics were extracted in terms of the isolation and insertion loss. Figure 2e,f shows the de‐embedded measurements of the EC‐LL MoS_2_ memristor in LRS and HRS. The devices have been characterized in two frequency bands: 10–43.5 and 67–110 GHz. The LRS shows an insertion loss of 0.42 dB at low frequencies. At higher frequencies, the insertion loss increases to an average of 0.9 dB but remains within acceptable limits as a performance metric for an RF switch. When the memristor device is switched to the reset state, the isolation is greater than 18 dB over all frequencies up to 110 GHz. While traditional transmission‐line RF switches often demand isolation values exceeding 25 dB to ensure signal purity, it is crucial to distinguish these requirements from those of switches embedded in RIS. In RIS technology, the memristor acts as a reconfigurable element to modify the reactance of the unit cell radiator, meaning the desired unit cell behavior is inherently co‐optimized around the switch's intrinsic isolation value. For context, mature commercial PIN diodes widely deployed in state‐of‐the‐art millimeter‐wave RIS and transmit‐arrays exhibit single‐device isolation levels that degrade to approximately 7.7–8 dB at frequencies between 30 and 60 GHz [46, 47, 48, 49, 50, 51]. When evaluated against these validated, high‐density spatial architectures, the >18 dB isolation achieved by our memristors is not merely acceptable, but highly competitive for millimeter‐wave integration. In addition, it was found that the EC‐LL MoS_2_ memristor achieves acceptable levels of reflectance coefficient over the entire frequency range, with values below −20 dB in the LRS state and above −1 dB in the HRS state. RF measurements have been performed on several devices in both states, and all provide stable S‐parameter measurements with low variation, see Note S3. In the same context, the intrinsic electrical parameters of a memristor device, namely, *R*_ON_, *R*_OFF_, and *C*_OFF_, are fundamental performance metrics [3, 7]. Since the de‐embedding process was performed on a 5 × 5 µm section and the memristor was only fabricated in a 2 × 2 µm section, the measured data represent a memristor device embedded in a small CPW section that was not de‐embedded. The de‐embedded measured devices can therefore be modelled as a lumped element model containing the equivalent circuit of a memristor between two transmission line circuit models, see Figure S11. The measurements were fitted to the circuit lumped model to determine the parameters of the circuit model and extract the intrinsic values of the memristor. The simulation results obtained from the fitting process were compared to the measured data and presented also in Figure 2e,f. It is worth noting that while the single equivalent circuit model shows excellent agreement at low frequencies, which is the primary operational band for our 27.5 GHz RIS application, there is an observable deviation in the fitting at high frequencies (>67 GHz), particularly in the HRS state. This discrepancy arises because the broadband measurements were conducted using two physically distinct experimental setups: a standard VNA configuration for the low band (up to 43.5 GHz) and a frequency‐extender configuration with a different RF probe set for the high band (67–110 GHz). The transition between these setups introduces varying test‐fixture parasitic capacitances that a single, unified equivalent circuit model cannot perfectly capture simultaneously across the entire 110 GHz spectrum. However, because the model accurately captures the baseline performance in our primary operational band, the extracted intrinsic values remain highly reliable for the subsequent RIS design. The intrinsic parameters of the EC‐LL MoS_2_ memristor are *R*_ON_ = 6.8 Ω, *R*_OFF_ = 14.1 kΩ, and *C*_OFF_ = 4.3 fF.

This OFF‐capacitance is exceptionally low compared to reports in literature on CVD MoS_2_; an idealized, perfectly flat 4 nm thick device of this area would yield a theoretical capacitance of approximately 28.3 fF. To understand this discrepancy, we investigated the physical morphology of the active layer. AFM (Figure 1d) and XPS reveal that residual PVP (*ε*_r_ = 3.5) [52, 53, 54] forms distinct nanoscale agglomerations on the MoS_2_ sheets (*ε*_r_ = 3.2) [15]. Rather than perfectly conforming to these polymer bumps, the continuous 2D MoS_2_ flakes bridge across adjacent peaks due to high in‐plane stretching stiffness and finite out‐of‐plane bending rigidity [55, 56]. This structural “tenting” traps microscopic interfacial air gaps beneath the active flakes. To quantify this effect, we developed a 3D statistical capacitance model based directly on the empirical AFM topography. Monte Carlo simulations demonstrate that these stochastically distributed air gaps (*ε*_r_ = 1) fundamentally alter the effective dielectric volume, reducing the theoretical flat‐layer capacitance by over 50% to a median of ∼11.3 fF. The remaining discrepancy to our experimental *C*_OFF_ (∼4.3 fF) is attributed to macro‐scale fabrication non‐idealities outside the scope of this localized model, such as reduced effective electrode overlap from lift‐off edge bending and microscopic discontinuities in the MoS_2_ flakes, as well as eventual zones with overlap of more than two nanosheets. Nonetheless, this qualitatively validates that the PVP‐induced “tenting” mechanism is a primary driver of the exceptionally low RF capacitance observed in these devices. Full details regarding the geometric extraction, statistical distributions, and simulation methodology are provided in Note S4 and Figure S12.

The memristor's cut‐off figure of merit (FOM) = (2π*R*_ON_ *C*_OFF_)^−1^ can be extracted to be higher than 5 THz. The switching ratio is also a key performance indicator, measuring the ratio of rejected signals to transmitted signals. As depicted in Figure 2g, the proposed memristor offers a switching ratio greater than 18 dB over the entire band. This represents an average 5 dB improvement in switching ratio performance compared to RF switches based on CVD‐MoS_2_ [7], while hBN [18] based memristors show better switching ratio performance. However, the reported hBN devices display smaller areas, which require costly and time‐consuming electron beam lithography.

Considering the comparatively large area, the metrics make the EC‐LL MoS_2_ memristor a promising candidate to be used as a high‐performance, but cost‐effective RF switch for various applications in the 6G communications, such as RIS and reconfigurable transmit arrays at microwave and mm‐wave frequencies.

A recognized challenge for 2D memristor‐based RF switches is the “self‐switching” phenomenon, where high incident RF power inadvertently triggers an unintended resistive state change. However, within the context of RIS, the input power is derived from an RF wave spatially impinging across a large aperture. Consequently, the actual RF power density distributed to any individual unit cell is exceedingly small, remaining well below the thermal or electrical thresholds required to induce a phase transition. To rigorously validate the power‐handling capabilities and resilience against self‐switching, *P*_IN _− *P*_OUT_ stress measurements were performed up to 40 GHz. The devices exhibited excellent power handling: a programmed LRS device (∼5 Ω) demonstrated a linear response with no measurable degradation or unintended state changes up to an input power of approximately 12 dBm (Figure S13a–f). Similarly, a device in the HRS (∼5.3 kΩ) maintained robust isolation without undergoing premature set events under the same stress conditions (Figure S13g–h). These results confirm that the devices possess sufficient power‐handling resilience to avoid self‐switching in spatial‐field RF applications.

### 2.4 Simulation of Reflective Intelligent Surfaces for Communication Systems

**FIGURE 3:**
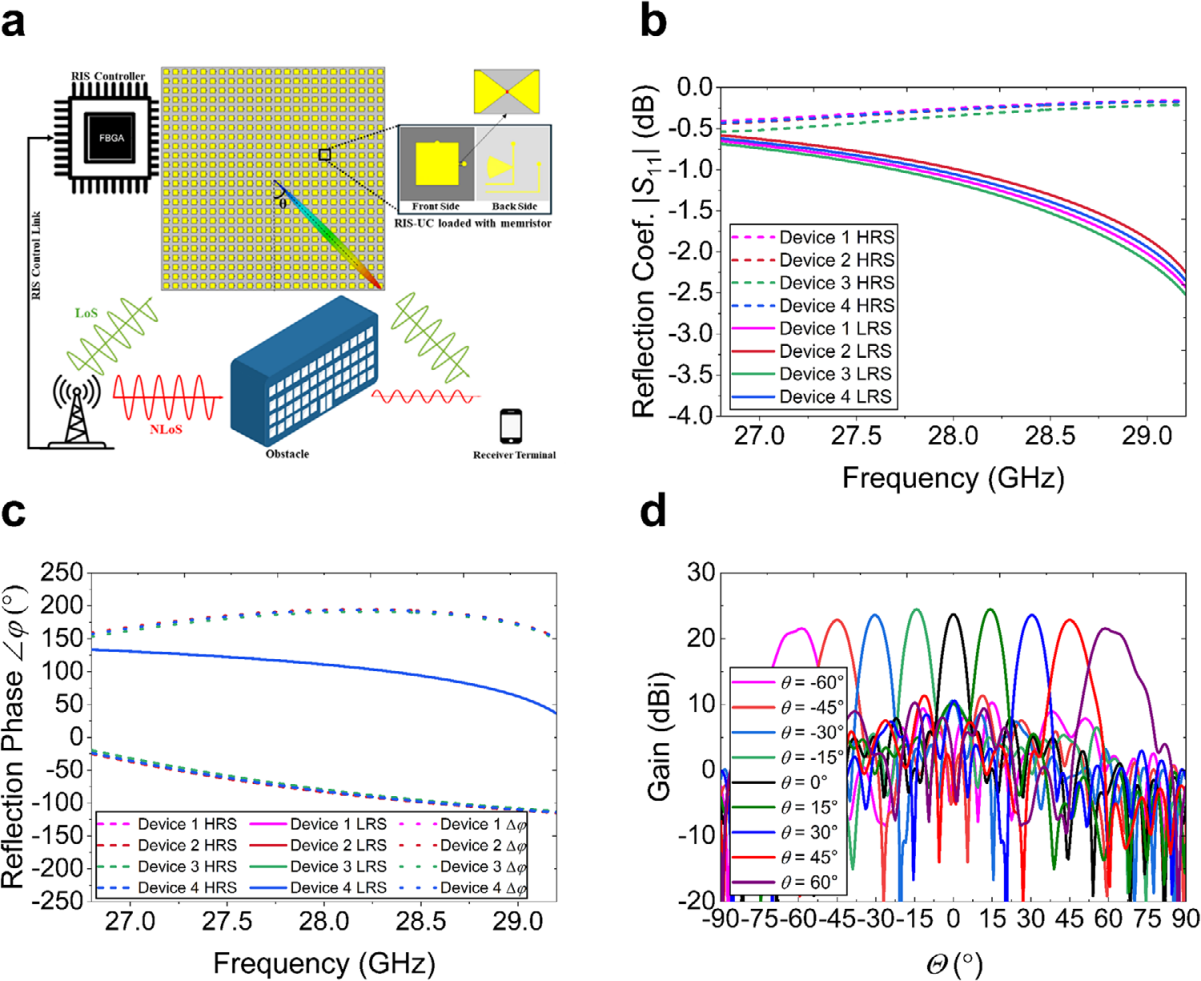
The simulated performance stability of the designed RIS panel based on EC‐LL MoS_2_ obtained measurement variability. (a) The beamforming capabilities offered by the 24 × 24 RIS. The electrical control of each unit cell's phase in the RIS enables the electromagnetic radiation steering such that the desired angle can be calculated based on the reflection phase distribution of all contributing unit cells in the array. The RIS UC performance stability according to the obtained measurements variability from four fabricated memristors: (b) Reflection coefficient magnitude |*S*_11_|, (c) Reflection coefficient phase for both switching states and phase difference (|Δ*φ*|). (d) The 2D far‐field radiation patterns for beam steering capabilities of the proposed RIS structure for scanning angles of *θ* = −60°: 60° spacing by 15° at frequency *f* = 27.5 GHz.

As previously mentioned, reconfigurable technology is a core component of RIS systems, playing a pivotal role in enhancing the energy efficiency of next‐generation communication networks. As shown in Figure 3a we present a simulation study for an RIS performance based on the memristor measurements to demonstrate the proficiency of EC‐LL MoS_2_ memristor parameters in achieving beamforming and beam scanning capabilities. In this regard, we designed and simulated a memristor‐based 1‐bit RIS unit‐cell (RIS‐UC) operating at Ka‐band. The design is presented in Note S5 and Figures S14 and S15. The reflective layer of the RIS unit cell consists of a square patch on a fused silica substrate, connected to the ground through a memristor. When an electromagnetic wave impinges on the unit cell, it induces a surface current density whose distribution is governed by the memristor state: in the HRS, the current is more uniformly distributed across the surface, whereas in the LRS, the current is effectively shorted to ground. The performance of the unit cell expressed in terms of reflection coefficient, both magnitude and phase, is shown in Figure S11. The simulated results show that the magnitude of the reflection coefficient |*S*_11_| remains above −2 dB across the 26.8–29.1 GHz frequency band for both states of the unit cell. In addition, considering a phase difference between the two states of the RIS element of |Δ*φ*| = 180° ± 20°, the unit cell achieved an operational bandwidth of approximately 2.2 GHz. The performance of the designed RIS‐UC was studied based on measurements obtained from four devices in order to investigate the stability of the RIS‐UC performance according to the variability of the intrinsic parameters of the memristors. Figure 3b,c shows the RIS‐UC performance represented by the reflection coefficient magnitude and phase. On average, the reflection coefficient of the RIS‐UC is affected by approximately 0.2 dB due to the variability of the memristor parameters. The reflection phase remains constant in the LRS state and is affected by approximately 3° in the HRS state of the memristor. However, a critical concern for the long‐term deployment of memristors in RIS is the reliability of the switch against natural, cycle‐to‐cycle variations in the *R_ON_* inherent to stochastic filament formation. To comprehensively evaluate the robustness of the device beyond the initial measured sample, a unit‐cell‐level analysis was performed to assess how the overall RIS performance responds to wider, expected *R*_ON_ operational variations ranging from 5 to 50 Ω. As illustrated in Figure S16a, the reflection coefficient magnitude is affected by this variation, with |*S*_11_| reaching approximately −6 dB at the primary frequency of 27.5 GHz for higher resistances. This reduction in unit cell reflection translates to a modest overall RIS gain penalty of 1–2 dB, an acceptable trade‐off given the architectural power savings. Crucially, as shown in Figure S16b, the reflection phase (∠*S*_11_) remains entirely constant across the full 5–50 Ω range. Similarly, to evaluate the impact of geometric randomness and surface roughness on functional yield in the OFF‐state, a stability analysis was conducted across an exaggerated range of *C*_OFF_ values from 0.5 to 6 fF. As shown in Figure S17, the effect of *C*_OFF_ variation on the reflection magnitude is negligible, with all |*S*_11_| values remaining below the −1 dB level. Furthermore, while this wide *C*_OFF_ variation introduces a maximum deviation of 13° in the reflection phase difference, this remains comfortably within the acceptable tolerance range for a 1‐bit RIS unit cell (Δ*φ* = 180° ± 20°). Consequently, because the reflection phase difference remains highly stable under experimental fabrication tolerances, simulated long‐term resistance fluctuations, and wide OFF‐state capacitance spreads, the correct operation of the RIS‐UC is not compromised. This proves that the designed RIS architecture is fundamentally robust against the inherent variability of the memristors.

A RIS array with an aperture size of 24 × 24 elements with dimensions of 9.5*λ*_0 _× 9.5*λ*_0_ × 0.07*λ*_0_ mm^3^ was designed and simulated. By controlling the phase distribution across the aperture, the RIS panel directs the radiation energy to specific regions of interest while suppressing interference from other directions. The beam steering capability of the designed RIS aperture is demonstrated across an angular range from *θ* = −60° to *θ* = 60° at a frequency of 27.5 GHz, as shown in Figure 3d. The simulated gain is plotted against the azimuthal angle (*Θ*) for the various steering configurations, illustrating the performance of the RIS. The achieved gain of the simulated RIS panel exceeds 21.6 dBi over all angles of observation angles, so that the gain of the generated main reflected beam ranges from 24.47 to 21.6 dBi for scan angles from 0 to ±60°. The simulation results show that at a reflection angle of *θ* = ±60°, there is a small scanning loss of 2.1 dBi compared to focusing the electromagnetic wave at an angle of *θ* = 0°. This is a realistic figure for the RIS performance due to the effective aperture reduction with the cosine of the observed angle. The electromagnetic radiation is precisely directed to the predetermined observed angles with an extremely low beam sequence. The maximum difference between the observed beam angle and the desired beam angle was 1.5° at *θ* = ±60°. It is worth noting that the minimization of the beam sequent has a positive effect on the reliability of the communication link and the suppression of interference levels. The aperture efficiency (*η*_ap_) measures how effectively a RIS panel utilizes its physical aperture area. It is calculated as *η*_ap_ = *Gλ* ^2^/(4π*A*_ap_), where *G* is the gain, *λ* is the wavelength, and *A*_ap_ is the physical area of the RIS. Based on the attained RIS gain, the maximum aperture efficiency is approximately 24.6% at *θ* = 15° while the aperture efficiencies achieved at *θ* = 0°, 30°, 45°, and 60° are 20.6%, 20.2%, 17% and 12.6%, respectively. The simulated gain versus frequency in Figure S11 shows a stable behavior of the designed RIS panel in terms of gain and aperture efficiency. Furthermore, to more clearly emphasize the efficiency and effectiveness of memristors in RIS performance as a reconfigurable technique, the gain and aperture efficiency of PIN‐based and ideal switch‐based RISs are compared with the memristor‐based RISs. An ideal RF switch has zero resistance and zero insertion loss when it is in the ON state. When it is switched to the OFF state, it provides infinite isolation and perfectly blocks the transmitted signal. To model these characteristics in the RIS panel, the modelled memristor is replaced by a metal short circuit when it is in the ON state and by an open circuit when it is in the OFF state. At an observation angle of *θ* = 15°, the RIS based on ideal switches demonstrated optimal performance, achieving a gain of 25.15 dBi and an aperture efficiency of 28.87%. Next came the memristor‐based RIS with 24.47 dBi and 24.6%, while the PIN diode‐based RIS performed worst with 23.3 dBi and 18.9%. These results demonstrate that memristors can enhance the efficiency of RF switches based on RIS technology, approaching ideal performance, besides their advantage of eliminating static power consumption typically associated with control boards, such as field‐programmable gate arrays and drive circuits.

## 3 Conclusion

This work highlights the promising performance and integration potential of EC‐LL MoS_2_ memristors as non‐volatile RF switches for next‐generation wireless systems. Utilizing an innovative fabrication approach that combines electrochemical exfoliation of MoS_2_ nanosheets with a liquid–liquid interfacial assembly technique, we demonstrate a scalable, solution‐based method for producing uniform, high‐quality 2D thin films with enhanced layer continuity, low porosity, and minimal contamination. This process enables the formation of densely packed bilayer MoS_2_ networks with superior electronic and morphological properties compared to conventional deposition techniques, directly contributing to the memristor's low insertion loss (<1 dB), high isolation (>18 dB), and ultra‐high cut‐off frequencies (>5 THz).

The resulting memristor devices exhibit robust unipolar switching, favorable intrinsic electrical parameters (*R*_ON_ = 6.8 Ω, *R*_OFF_ = 14.1 kΩ, *C*_OFF_ = 4.3 fF), and stable RF performance across a wide frequency range, making them highly suitable for mmWave applications. Their non‐volatile behavior is particularly advantageous for power‐constrained systems like RIS, where energy consumption is a critical bottleneck. A full RIS array simulation confirms high gain and precise beam directionality, positioning this technology as a leading solution for 6G communication infrastructures. The experimental device‐to‐device variability was confirmed to have no significant effect on the simulated RIS performance.

Future work will focus on mechanistic studies of the switching process, optimizing interfacial engineering, and statistically analyzing device uniformity across large batches. These efforts will drive the transition from proof‐of‐concept to industrially viable, high‐performance RF switches and programmable metasurfaces. In doing so, memristor‐based nv‐RS devices stand poised to meet the ambitious goals of the 6G era—delivering compact, low‐power, and reconfigurable systems for a more connected and sustainable future.

## 4 Experimental Section/Methods

### 4.1 Synthesis of 2D Material Dispersion

MoS_2_ nanosheets were prepared using an electrochemical intercalation method. A two‐electrode setup with a graphite foil anode and MoS_2_ single‐crystal (natural origin, Krupka, Czech Republic) cathode was immersed in 40 mL of electrolyte containing 12.5 mg/mL tetraheptylammonium bromide in acetonitrile. A 7 V potential was applied for 1 h, leading to MoS_2_ expansion. The intercalated MoS_2_ was rinsed with acetone and sonicated in a 10 mg/mL solution of polyvinylpyrrolidone (PVP) in dimethylformamide (DMF) for 30 min.

Cascade centrifugation refined the nanosheet thickness distribution. Large nanosheets were removed by centrifugation at 958 g for 30 min, followed by a second step at 3830 g for 60 min to eliminate smaller sheets. The sediment from the second centrifugation step was redispersed in IPA to obtain the final MoS_2_ dispersion.

### 4.2 Synthesis of MoS_2_ Monolayers

A liquid‐interface assembly method previously described in the literature [37, 38] was used to deposit monolayer networks of MoS_2_. Hundred milliliters of deionized water (>18 MΩ cm) and 50 mL of *n*‐hexane (Sigma Aldrich) were added to a 200 mL beaker. A substrate with patterned bottom electrodes was placed on a homebuilt stage and submerged beneath the water–hexane interface using a dip coater (Ossila). MoS_2_ dispersions were injected at 150 µL/min using a syringe pump (ISPLab01, Infusetek). Stirring of the water phase at 100 rpm using a magnetic stirrer ensured spatial uniformity of the assembled MoS_2_ monolayer. After complete monolayer formation, stirring was ceased, and the substrate was lifted vertically through the interface at 1 mm/s to transfer the monolayer. This deposition process was repeated to produce multilayer networks. This process was carried out at the School of Physics, CRANN & AMBER Research Centers, Trinity College Dublin, Ireland, which required the devices to be shipped there after the bottom electrodes’ deposition and back for the top electrodes.

### 4.3 Device Fabrication

The RF switches were fabricated on glass substrates that were previously cleaned in subsequent ultrasonic baths of acetone and isopropanol, followed by rinsing with deionized water and dry nitrogen. The glass substrate used was 1 mm thick 7980 fused silica, which was chosen due to its dielectric constant. *ε*_r_ = 3.81 and loss tangent, tan *δ* = 0.0004. The bottom electrodes were comprised of a 10 nm Ti adhesion layer and 60 nm Au deposited by e‐beam evaporation. The top electrodes were also deposited by e‐beam evaporation, a 2 Cr adhesion layer, and 70 nm Au. Both were patterned by photolithography and lift‐off.

### 4.4 RF Test Fixtures

To enable the high‐frequency characterization, a GSG waveguide test fixture was deposited on top of the individual switches. For these, a ∼700 nm thick Cu layer was deposited by e‐beam and patterned by photolithography and lift‐off, see Note S6 and Figure S18.

### 4.5 Electrical Characterization

DC measurements were carried out using a Keithley 4200 SCS semiconductor analyzer connected to a Janis ST‐500 probe station Figure S19. The bias was applied to the top electrode, with the bottom electrode grounded. The measurement speed was set to normal mode, with integration time in auto‐setting, allowing the system to optimize based on range and other conditions. Both hold and delay times were set to zero. Sweeps were performed in a one‐way direction, always beginning at 0 V, with a cease test trigger at compliance. The reset procedure involved two stages: an *I*–*V* sweep from 0 to 0.5–0.7 V with a current compliance of 50–80 mA, followed by a sweep from 0.5–0.7 to 1.2–1.4 V with a compliance of 1–5 mA, adjusted for device‐to‐device variations. For the set operation, a current‐controlled sweep was applied from 1 µA to 5 mA.

### 4.6 RF Characterization

High‐frequency broadband RF measurements covering the 10–110 GHz spectrum were conducted in distinct frequency bands (10–43.5 and 67–110 GHz). This split‐band approach was necessitated by the physical limitations of the probing equipment, requiring a transition between different RF probe sets and waveguide frequency extenders to fully bridge the wide spectrum. For further RF measurements methodology details, please check Note S2.

### 4.7 Simulation Methodology

Electromagnetic simulations of the RIS‐UC arrays and test fixtures were performed using CST Studio Suite. To evaluate the RIS‐UC performance, unit cell (periodic) boundary conditions were applied to the lateral boundaries to emulate an infinite array environment, while Floquet ports were utilized to model the incident and reflected waves and extract the reflection characteristics. For the full RIS array simulations, the structure was evaluated in free space, illuminated by a horn antenna, and its far‐field radiation characteristics were analyzed to estimate the overall RIS gain across different array reconfiguration states. Additionally, the equivalent circuit modeling based on the de‐embedded measurements was carried out using Keysight ADS. Further details on the physical simulation stack‐up are provided in Note S5.

## Author Contributions

L.M.P., A.K., and J.D. have designed the project. T.M. and J.N. have fabricated the samples. Z.S. has provided the MoS_2_ crystals. T.M., M.E.G., L.M., and S.M. have performed sample characterization. T.M., L.M., M.E.G., S.M., L.L, and A.C. have analyzed the data. T.M., M.E.G., A.K., and J.D. wrote the article. J.M.C., J.V., L.M.P., E.F., and R.M. have provided access to the required facilities. All authors discussed the results and revised the manuscript.

## Funding

This work has been supported by the Smart Networks and Services Joint Undertaking (SNS JU) under the European Union's Horizon Europe research and innovation programme under Grant Agreement No. 10109710 (TERRAMETA). Furthermore, this work was financed by national funds from FCT ‐ Fundação para a Ciência e a Tecnologia, I.P., in the scope of the projects LA/P/0037/2020, UIDP/50025/2020 and UIDB/50025/2020 of the Associate Laboratory Institute of Nanostructures, Nanomodelling and Nanofabrication – i3N. T.M. acknowlegdes FCT for the doctoral grant 2024.04237.BD. J.N. acknowledges funding from ADMIRE Marie Skłodowska‐Curie COFUND Postdoctoral Fellowship “SLAM‐2D” (grant number 12/RC/2278_P2). Z.S. was supported by project LUAUS25268 from the Ministry of Education, Youth and Sports (MEYS) and by the project Advanced Functional Nanorobots (reg. No. CZ.02.1.01/0.0/0.0/15_003/0000444 financed by the EFRR). A.G.K. acknowledges funding from the European Commission through a Marie Skłodowska–Curie Postdoctoral Fellowship “NanoHarvest” (proposal number: 101107032). L.M. and J.C.V. acknowledge funding by FCT/MECI through national funds and, when applicable, co‐funded EU funds under UID/50008: Instituto de Telecomunicações.

## Conflicts of Interest

The authors declare no conflicts of interest.

## Data Availability

The data that support the findings of this study are available from the corresponding author upon reasonable request.
